# Deep Transcriptomic Profiling of M1 Macrophages Lacking Trpc3

**DOI:** 10.1038/srep39867

**Published:** 2017-01-04

**Authors:** Sivarajan Kumarasamy, Sumeet Solanki, Oluwatomisin T. Atolagbe, Bina Joe, Lutz Birnbaumer, Guillermo Vazquez

**Affiliations:** 1 Department of Physiology and Pharmacology, and Center for Hypertension and Personalized Medicine, University of Toledo College of Medicine and Life Sciences, University of Toledo Health Science Campus, 3000 Transverse Dr., Toledo, Ohio 43614 USA; 2 Neurobiology Laboratory, National Institute of Environmental Health Sciences, 111 TW Alexander Dr., Research Triangle Park, North Carolina 27709 USA; 3 Institute of Biomedical Research (BIOMED UCA-CONICET), Faculty of Medical Sciences, Av. Alicia Moreau de Justo 1600, C1107AFF Buenos Aires, Argentina

## Abstract

In previous studies using mice with macrophage-specific loss of TRPC3 we found a significant, selective effect of TRPC3 on the biology of M1, or inflammatory macrophages. Whereas activation of some components of the unfolded protein response and the pro-apoptotic mediators CamkII and Stat1 was impaired in Trpc3-deficient M1 cells, gathering insight about other molecular signatures within macrophages that might be affected by Trpc3 expression requires an alternative approach. In the present study we conducted RNA-seq analysis to interrogate the transcriptome of M1 macrophages derived from mice with macrophage-specific loss of TRPC3 and their littermate controls. We identified 160 significantly differentially expressed genes between the two groups, of which 62 were upregulated and 98 downregulated in control *vs.* Trpc3-deficient M1 macrophages. Gene ontology analysis revealed enrichment in processes associated to cellular movement and lipid signaling, whereas the enriched Kyoto Encyclopedia of Genes and Genomes (KEGG) pathways included networks for calcium signaling and cell adhesion molecules, among others. This is the first deep transcriptomic analysis of macrophages in the context of Trpc3 deficiency and the data presented constitutes a unique resource to further explore functions of TRPC3 in macrophage biology.

Transient Receptor Potential Canonical 3 (TRPC3) is a non-selective Ca^2+^-permeable channel that belongs to the TRPC family (TRPC1-7) within the larger TRP superfamily of cation channels^1^,^2^. Under physiological conditions TRPC3 is regulated by receptor stimulation of diacylglycerol-producing phospholipases and exhibits receptor-independent or constitutive function^3^. In previous studies from our laboratory using a bone marrow transplantation strategy as a first approach to examine a potential role of the macrophage Trpc3 in atherosclerosis, we found that the advanced aortic plaques of hyperlipidemic mice with bone marrow-selective deletion of Trpc3 have less necrosis and reduced number of apoptotic macrophages than control animals, parameters usually indicative of more stable plaques^4^. In more recent studies using macrophages derived from mice with macrophage-specific loss of TRPC3 function and differentiated *in vitro* to the M1 and M2 types, we observed that lack of Trpc3 reduces activation of the unfolded protein response (UPR) with a consequent decreased susceptibility to endoplasmic reticulum (ER) stress-induced apoptosis, providing a potential explanation to the *in vivo* findings^5^. Remarkably, this effect was selective for M1 macrophages, as genetic or pharmacological inhibition of Trpc3 reduced activation of the UPR and ER stress-induced apoptosis in M1, but not M2 macrophages^5^. In that study, we also showed that the lack of Trpc3 impaired the functions of calmodulin-dependent protein kinase II and Stat1 only in M1 macrophages. Considering that TRPC3 is a calcium-permeable channel, evaluating the impact of TRPC3 expression on signaling molecules, whose performance depends, directly or indirectly, upon calcium influx into the cell seemed a logical approach. However, gathering insight on molecular signatures within macrophages that might be specifically affected by TRPC3 requires an alternative tactic. In this context, an unbiased genome-wide approach provides a more powerful strategy^6^. In the present study we conducted RNA-seq analysis to interrogate the whole transcriptome of M1 macrophages derived from mice with macrophage-specific loss of Trpc3 function or their littermate controls. The data obtained is of specific value and provides information on global signatures to understand the contributions of coding and non-coding RNAs that may exert an effect in shaping the macrophage transcriptome pathways, and on potential molecular players whose expression/function is affected by Trpc3.

## Results

**Figure 1:**
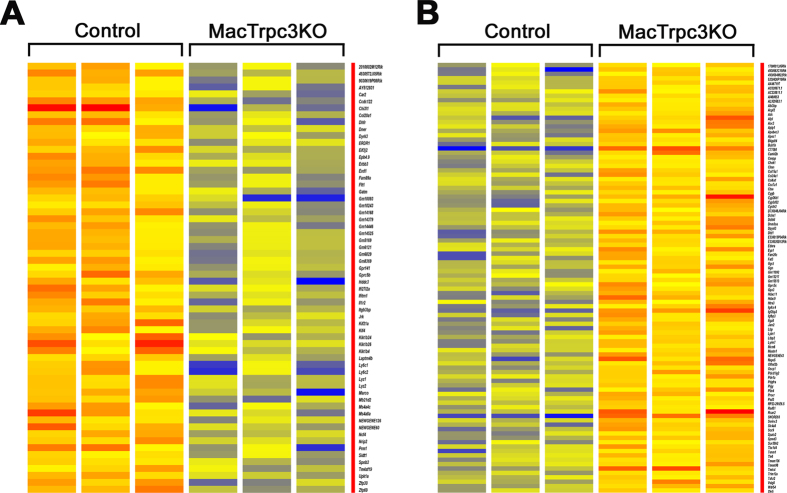
Heat map of differentially expressed transcripts ordered alphabetically. Transcripts with statistically significant alterations (p < 0.05) in expression between control (TRPC3-expressing, n = 3) and MacTRPC3KO (TRPC3-deficient, n = 3) M1 macrophages. A: genes upregulated in control *vs.* MacTRPC3KO; B: genes downregulated in control *vs.* MacTRPC3KO. Intensities are normalized for each differentially expressed (2.0-fold up/down; p < 0.05) from RNA sequencing data. Each column represents 1 of 3 RNA samples, *i.e.*, biological replicates for each genotype.

To obtain unbiased insight on molecular signatures within M1 macrophages that might be specifically affected by the loss of Trpc3 expression, we performed deep RNA sequencing (RNA-seq) analysis of RNA samples of M1 macrophages derived from bone marrows of mice with macrophage-specific loss of Trpc3 function (MacTRPC3KO, or Trpc3-deficient cells) or their littermate controls (TRPC3^lox/lox^, controls or TRPC3-expressing cells). Following *in vitro* interferon γ-induced differentiation of bone marrow-derived macrophages to the M1 type (as we described in^4^,^5^,^7^), RNA was isolated and subjected to RNA-seq analysis. The average paired-end sequencing reads per individual library was 110.2 million, with 97% of read alignment. RNA-sequencing detected 14,605 and 14,867 transcripts in TRPC3^lox/lox^ and MacTRPC3KO M1 macrophages, respectively. This comprised transcripts for both protein-coding genes and non-coding RNAs. After gene expression quantification with DESeq normalization, an unpaired *t-*test was carried out to compare sequence reads derived from control (n = 3) *vs.* Trpc3 deficient (n = 3) M1 macrophages. This comparison identified 160 significantly differentially expressed genes (fold change ≥2.0, p < 0.05) between the two groups (Tables S2 and S3). Among these, 62 transcripts were upregulated in control *vs.* MacTRPC3KO M1 macrophages (range + 2.0 to 10.54 fold; Fig. 1A), whereas 98 transcripts were downregulated in controls compared to the TRPC3-deficient M1 cells (range −2.0–17.1 fold; Fig. 1B).

**Figure 2:**
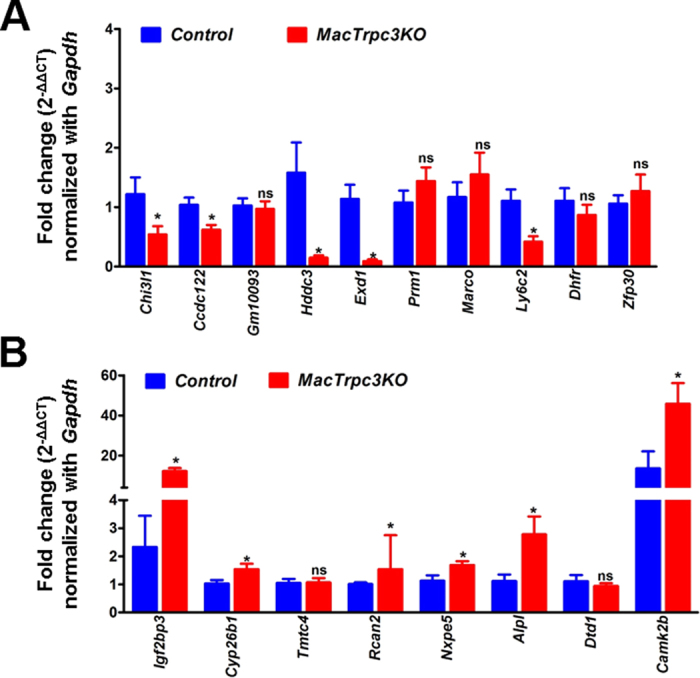
Validation analysis of select RNAseq data with qRT-PCR. The top 10 genes that exhibited highest fold change in differential expression between control and MacTRPC3KO M1 macrophages (Tables S4 and S5) were evaluated by qRT-PCR as described in Methods. **A)** qRT-PCR of the top 10 genes with highest fold change in upregulated expression in control *vs.* MacTRPC3KO macrophages. **B)** qRT-PCR of the top 8 genes with highest fold change in downregulated expression in control *vs.* MacTRPC3KO macrophages. *C77080* and *Apobec3* (Table S3) produced inconsistent results and are not included in the figure. Data were normalized to *Gapdh* expression and fold-change in expression was calculated by the 2^−ΔΔCT^ method. *p < 0.05; ns: not statistically significant.

**Figure 3:**
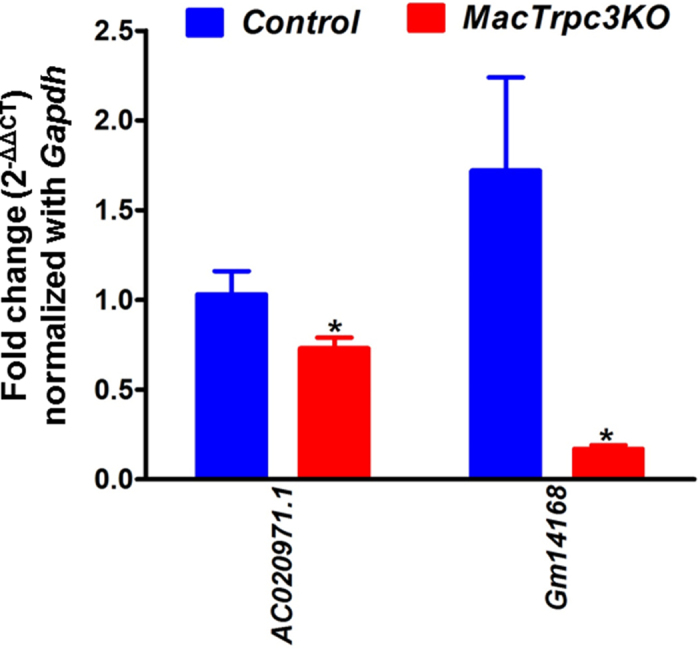
qRT-PCR analysis of lincRNAs*AC020971.1* and *Gm14168* which were prominently differentially expressed in control *vs.* MacTRPC3KO M1 macrophages (Tables S2 and S3). Data was normalized to *Gapdh* expression and fold-change in expression was calculated by the 2^−ΔΔCT^ method. *p < 0.05; ns: not statistically significant.

We next selected the top ten transcripts with highest fold change in expression (upregulated or downregulated) between Trpc3-expressing and Trpc3-deficient M1 macrophages for further validation by qRT-PCR (up- and down-regulated genes, Tables S4 and S5). Among these, the expression of *Chi31l, Ccdc122, Hddc3, Exd1* and *Ly6c2* were significantly elevated in control macrophages compared to Trpc3-deficient M1 cells (Fig. 2A), whereas *Igf2bp3, Cyp26b1, Rcan2, Nxpe5, Alpl* and *Camk2b* were confirmed as significantly downregulated in controls compared to Trpc3-deficient M1 cells (Fig. 2B). We also examined the expression level of 7 long non-coding RNAs (LincRNA, fold change ≥2.0; Tables S2 and S3). The results from qRT-PCR analysis showed that LincRNAs *AC020971.1* and *Gm14168* were downregulated in TRPC3-deficient M1 macrophages compared to controls (Fig. 3). In addition, 195 novel genes –*i.e.*, new gene annotations with no knowledge of whether they are coding or non-coding RNAs- (Table S6) were detected in samples from both control and Trpc3-deficient M1 cells, as well as 21 differentially spliced transcripts between Trpc3-expressing and Trpc3-deficient M1 macrophages (Table S7).

We next conducted a web based gene ontology (GO) analysis to identify the biological processes that were significantly enriched based on those transcripts that were significantly differentially expressed between control and MacTRPC3KO M1 macrophages. The top ten GO enriched biological processes are listed in Table S8. A majority of processes were associated to cellular movement (cell migration, motility, locomotion, movement) and lipid signaling (PI3-kinase and lipid kinase activities, inositol lipid and phosphinositol signaling), all with ≥2.0 fold change (p < 0.05). In addition, Table S9 shows the enriched Kyoto Encyclopedia of Genes and Genomes (KEGG) pathways associated with transcripts that exhibited significantly reduced expression in control M1 macrophages compared to the TRPC3-deficient cells. These included, among others, calcium signaling, cell and focal adhesion molecules and actin cytoskeleton. Amongst the enriched KEGG pathways associated to transcripts with significant upregulation in control M1 macrophages compared to TRPC3-deficient cells, were those for salivary secretion, cytokine-cytokine receptor interaction, endocytosis, phagosome and metabolic pathways (Table S10).

**Figure 4:**
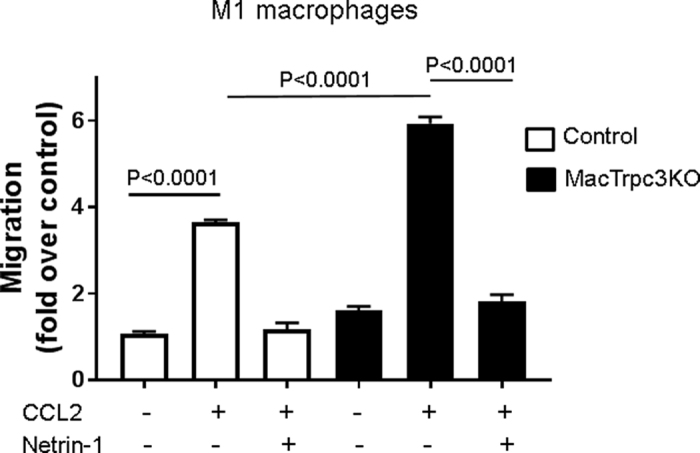
M1 macrophages prepared from MacTRPC3KO or littermate control mice (control) were suspended in complete medium and added (10^6^/ml) to the upper compartment of 96-well Boyden chambers (5 μm pore), in the presence or absence of CCL2 (80 ng/ml) in the lower compartment, and with or without pre-treatment with mouse recombinant netrin-1 (40 min, 200 ng/ml). After 12 h, cells that migrated to the lower compartment were counted (5 random fields/well, triplicate wells/condition). Shown is fold change over controls (# migrating cells in condition “x”/# migrating cells in control).

Since pathway analysis identified a number of processes associated to cell motility that were upregulated in Trpc3-deficient M1 macrophages, we next wished to examine whether Trpc3 deficiency had indeed an impact on the ability of macrophages to migrate in response to a chemokine. The data in Fig. 4 shows that M1 macrophages with loss of TRPC3 function exhibit increased migration in response to CCL2 compared to Trpc3 expressing M1 cells. As expected, CCL2-induced migration of M1 macrophages was almost completely prevented by pre-treatment with the retention cue netrin-1, regardless of Trpc3 expression.

## Discussion

Previous work from our group demonstrated that in macrophages the TRPC3 channel has a pro-apoptotic role, as evidenced by reduced necrosis and number of apoptotic macrophages in the advanced atherosclerotic plaques of a hyperlipidemic mouse model of atherosclerosis with bone marrow-selective deletion of Trpc3^4^. Additional *in vitro* studies in polarized macrophages derived from mice with macrophage-specific loss of TRPC3 function showed that the effects of TRPC3 were selective for the M1, or inflammatory macrophages, with no impact on the M2, or anti-inflammatory type^5^. *A priori*, identifying global alterations in signaling candidates downstream of Trpc3 that might be affected by the loss of channel function is not feasible from a canonical biochemical/cell biology stand point, considering the myriad of cellular processes that may be affected, directly or indirectly, by Trpc3. Thus, gathering insight on molecular signatures within macrophages that might be specifically affected by the lack of Trpc3 requires a different strategy. The deep transcriptome profiling performed in this study was aimed at obtaining detailed, unbiased information on global transcriptomic signatures in Trpc3 deficient M1 macrophages. Aside from generating a genome-wide transcriptome blueprint as a result of lack of Trpc3, the goal was also to uncover previously unknown and thereby underappreciated contributions of non-coding RNAs in shaping transcriptome pathways in inflammatory M1 macrophages. Applying a stringent detection threshold (>2.0-fold change) and a cut-off *p*-value of <0.05, we identified statistically significant changes in the expression levels of 160 genes between Trpc3-expressing and Trpc3-deficient M1 macrophages. This revealed the existence of differentially expressed transcripts for protein-coding RNAs, non-coding RNAs and new genes in M1 macrophages with loss of TRPC3 function, when compared to control cells. Among the non-coding RNAs, RNA-seq analysis revealed 7 long non-coding RNAs (lincRNAs) with differential expression between control and Trpc3-deficient M1 macrophages. Two out of this 7 lincRNAs, lincRNAs *AC020971.1* and *Gm14168* were prominently downregulated in macrophages with loss of Trpc3 function compared to Trpc3-expressing cells. LincRNA AC020971.1 has been reported to be upregulated in epithelial cells from mouse lenses^8^, and *Gm14168* is a predicted annotation with little experimental validation. The targets and cellular functions of both of these LincRNAs remain unknown.

To gain insight into potential biological processes and molecular pathways affected by loss of Trpc3 in M1 macrophages, we evaluated the differentially expressed genes by using both gene ontology (GO) and KEGG. The GO analysis showed enrichment in several biological processes associated to cellular movement and lipid signaling. Although our previous studies focused on effects of TRPC3 in macrophage apoptosis, the changes that were revealed here in these other pathways are likely to contribute to other phenotypic features of M1 macrophages with loss of Trpc3 function. The changes observed in expression levels of several genes associated with cell movement and locomotion suggest the existence of alterations in motility pathways in TRPC3-deficient M1 macrophages. This contention is supported by functional data from an *in vitro* migration assay showing that Trpc3-deficient M1 macrophages have increased migratory response to CCL2. This is of interest, as migration and motility are critical functions of macrophages in the setting of atherosclerosis^9^,^10^,^11^. Changes in genes associated to cell migration have also been observed in other models of altered TRPC3 expression. For example, increased migration has been associated to augmented expression of TRPC3 in monocytes derived from patients with essential hypertension^12^. A recent microarray-based transcriptomic analysis of mouse Purkinje cells carrying the *Moonwalker* gain-of-function point mutation in Trpc3 revealed several biological pathways and functions that were significantly enriched in gene categories including lipid metabolism and cellular assembly and organization^13^. Worth noting among those genes showing upregulated expression in Trpc3-deficient M1 macrophages, are *Rcan2* and *Camk2b. Rcan2* (regulator of calcineurin) is a calcineurin-interacting protein that inhibits the phosphatase activity of calcineurin in several cell types^14^. M1 macrophages with loss of Trpc3 show increased levels of phospho-AKT, a key survival molecule in these cells (Solanki and Vazquez, unpublished observations), and calcineurin-mediated dephosphorylation of Akt is a widespread negative regulatory mechanism of this survival pathway^15^. Thus, the findings from the transcriptomic analysis point to *Rcan2* upregulation in Trpc3-deficient M1 macrophages as a potential mechanism underlying the decreased susceptibility of these cells to apoptosis^5^. In M1 macrophages TRPC3 function is coupled to tonic activity of CAMKII, and genetic or pharmacological inhibition of Trpc3 indeed impairs activation of this kinase^5^. In this context, it is likely that the marked upregulation of *Camk2b* in TRPC3-deficient macrophages may represent an attempt to compensate for such uncoupling. Among those transcripts with prominent downregulated expression in Trpc3-deficient M1 macrophages, it is worth noting that both *Chi2l1* and *Ly6C2*, which code for chitinase 3-like 1 and lymphocyte antigen 6 complex locus C2, respectively, are associated to a variety of inflammatory processes in both infectious and non-infectious diseases^16^,^17^. It remains to be determined whether downregulation of these genes has any effect on the inflammatory phenotype of M1 macrophages with Trpc3 deficiency^4^,^5^.

In sum, the present studies represent the first transcriptomic analysis of macrophages with loss of TRPC3 and identify alterations in a number of molecular pathways, many of which have not been previously linked to Trpc3. This information thus represents a unique resource for future studies aimed at identifying novel functions of Trpc3 in the context of macrophage biology, and to reveal whether alterations in these pathways could be targeted to modulate diseases with prominent macrophage involvement.

## Methods

### Experimental animals

All animal studies described in this work conform to the Guide for Care and Use of Laboratory Animals published by the NIH and have been approved by the University of Toledo Institutional Animal Care and Use Committee. Generation and characterization of LysMCre ^+/−^/Trpc3^lox/lox^ mice (for simplicity, MacTRPC3KO) was described in detail in^5^.

### Preparation of bone marrow-derived macrophages

Culture of bone marrow-derived macrophages, *in vitro* differentiation to the M1 type and phenotypic marker profiling of M1 cells was performed as we described in^4^,^5^,^7^,^18^. Importantly, Trpc3 deletion does not affect either macrophage maturation or their differentiation to the M1 type^4^,^5^.

### RNA library construction, sequencing and data analysis

Total RNA was extracted using 5 PRIME PerfectPure™ Purification System (5prime) before dispatch to Expression Analysis (EA) Sequencing & Bioinformatics (Durham, NC; www.ExpressionAnalysis.com). RNA quality was assessed by electrophoresis and bioanalyzer prior to RNA sequencing (Supplemental Fig. 1). An input of 100 ng of total RNA was used to construct cDNA libraries (TruSeq Stranded mRNA Sample Prep Kit, Illumina, #RS-122-2103) following the manufacturer’s instructions. Deep sequencing was done using the Illumina High Seq 2500 platform.

For RNA-seq analysis, low quality reads were removed and the adapter sequences were trimmed. The resulting sequences were mapped to the mouse reference genome (*MM10*, Ensemble genes and transcripts) using the Strand NGS software (Strand Life Sciences, version 2.1) following RNA alignment and RNA-seq analysis pipeline with standard parameters. Only those reads with high quality scores were retained for further analysis. Sequences aligned with individual transcripts were counted digitally. Differential gene expression was carried out with DESeq v.3.0 normalization to facilitate the comparison of transcripts among samples with fold change >2.0 and applying the Benjamini-Hochberg multiple test correction at a false discovery rate of 5% (adjusted p value <0.05). Gene annotations were provided by NCBI Entrez Gene database. Enrichment analysis of genes that were over- or under-represented was performed by Gene Ontology (GO) analysis^19^. Further analysis of biological pathways and molecular networks was conducted with the Kyoto Encyclopedia of Genes and Genomes (KEGG)^20^,^21^.

### Reverse transcription and real-time PCR

Total RNA was extracted using 5 PRIME PerfectPure™ Purification System (5prime). RNA samples were quantitatively analyzed using a NanoDrop ND-1000 to check the purity and concentration of the samples. One μg of total RNA was converted to cDNA using the superscript-III first strand synthesis kit (Invitrogen). The ABI 7300 Real Time PCR System with Power SYBR Green PCR Master Mix (Life Technologies) was used to carry out quantitative reverse transcription polymerase chain reaction (qRT-PCR) in duplicates. Gene expression levels were normalized with *Gapdh*, and the changes in expression were calculated by the 2^−ΔΔCT^ method as in^22^. Primer sequences are provided in Table S1.

### *In vitro* migration assay

this was performed essentially as described in^23^. M1 macrophages derived from bone marrows of MacTRPC3KO mice or their littermate controls were suspended in complete medium and added (10^6^/ml) to the upper compartment of 96-well Boyden chambers (5 μm pore). The lower compartment of the chambers contained the chemoattractant CCL2 (80 ng/ml). Alternatively, M1 macrophages were incubated with mouse recombinant netrin-1 (40 min, 200 ng/ml) before being added to the wells. The migration proceeded for 12 h, and at the end of this period migrated cells were counted (5 random fields/well, each condition run in triplicate). Results were expressed as fold change over controls (number of migrating cells in condition “x”/number of migrating cells in control).

### Statistical analysis

Statistical differences were determined using the Student’s *t-*Test and statistical significance was set at p < 0.05. Data are expressed as mean ± SEM.

## Additional Information

**Accession codes:** The RNA-seq data presented in this manuscript has been deposited in the NCBI GEO database (GSE8478).

**How to cite this article**: Kumarasamy, S. *et al*. Deep Transcriptomic Profiling of M1 Macrophages Lacking Trpc3. *Sci. Rep.* **7**, 39867; doi: 10.1038/srep39867 (2017).

**Publisher's note:** Springer Nature remains neutral with regard to jurisdictional claims in published maps and institutional affiliations.

## Supplementary Material

Supplementary Information
