# Supplementary material for: Deep Transcriptomic Profiling of M1 Macrophages Lacking Trpc3
Source: Sci Rep. 2017 Jan 4;7:39867. doi: 10.1038/srep39867 (PMC5209678; doi:10.1038/srep39867)

Supplementary information

DEEP TRANSCRIPTOMIC PROFILING OF M1 MACROPHAGES LACKING TRPC3

Sivarajan Kumarasamy, Sumeet Solanki, Oluwatomisin T. Atolagbe, Bina Joe, Lutz Birnbaumer and Guillermo Vazquez.

Table S1. **Primers used in qRT-PCR assays.**

S.NO	Primer Name	Primer Sequence	Product Size(bp)
1	*Gm14168-RT-F*	GAGGAGGAGGGAATTCTTGG	118
	Gm14168-RT-R	GCCAGCCTGGTCTACATAGC	
2	*AC020971.1-180922-F*	CAACAGTTCCGGAAGGAGAA	195
	AC020971.1-180922-R	TTGTAACTCGCCAGTCGTTG	
3	*Cyp26b1-RT-F*	ACTGGTTGCTACAGGGTTCC	14
	Cyp26b1-RT-R	GTGTTCGCCCAGTAGGATCT	
4	*Nxpe5-RT-F*	CCCTGTTATTGGCTGTCACT	129
	Nxpe5-RT-R	AATGGATCATGGGCTCAGG	
5	*Alpl-RT-F*	ATCAGCTAATGCACAATATCAAGG	101
	Alpl-RT-R	ATCCAGTTCGTATTCCACATCAGT	
6	*Camk2b-RT-F*	AATGTCTGAAGAAGTTCAATGCAA	117
	Camk2b-RT-R	CTCCATCTGCTTTCTTGTTGAGTA	
7	*Rcan2-RT-F*	GTGTTTGAAGAAGAGGAGAGCAA	96
	Rcan2-RT-R	GATTTGGGATGGCTGAAATTTAT	
8	*Tmtc4-RT-F*	CACTGAGTGTGTTGCTGGTG	138
	Tmtc4-RT-R	ACCCAGAAGGTGGAAGAATG	
9	*Hddc3-RT-F*	TGACAAGACTCTGCCCAAAC	150
	Hddc3-RT-R	TGTTCTGACCATCCTGTAGGG	
10	*Exd1-RT-F*	AGAGGAAGAGGAGGTGACATAC	144
	Exd1-RT-R	AGCCAACAGAGTTTCCCATG	
11	*Prm1-RT-F*	GAGGAGGCGAAGATGCTG	141
	Prm1-RT-R	TTTCAAGATGTGGCGAGATG	
12	*Dtd1-RT-F*	GAGCTGCTAGCTCTGACCCAAAG	168
	Dtd1-RT-R	GCTCAGAAGACACGTCACCCTC	
13	*Igf2bp3-RT-F*	AGT TCA AGG CTC AGG GAA GA	136
	Igf2bp3-RT-R	TTG CCT CCT TTC CCA ATA AC	
14	*Chi3l1-RT-F*	AGA CAA GCA GTA TTT CTC CAC CCT GAT C	139
	Chi3l1-RT-R	GAT GTC ATA GCC AGT GTC AAT GGC C	
15	*Ccdc122-RT-F*	TGT AGA GCA AGT CGC CAA ACA ACA AC	189
	Ccdc122-RT-R	TGA AGT TTA GAG TTC TCC ATT GCA GCA TCT TG	
16	*Gm10093-RT-F*	GCC CTA CAA CGA CTA CTT TGA ATA CT	103
	Gm10093-RT-R	GAT CTT CTC CAG GTA CTC GTT AGT GT	
17	*Marco-RT-F*	GAT TGC AGG TGT GAA AGG AAG CAA GGG TG	159
	Marco-RT-R	TTC TGA CCG ACT CGT CCA GGT TCT CC	
18	*Ly6c2-RT-F*	GTG CCA ATT GAG ACT TCC TGC CCA GCA G	140
	Ly6c2-RT-R	CCA GCA GGG CAG AAA GAA AGG CAC TGA	
19	*Dhfr-RT-F*	ACC TGG TTC TCC ATT CCT GAG AAG AAT C	165
	Dhfr-RT-R	GTC TAC TTT ACT TGC CAA TTC CGG TTG TTC	
20	*Zfp30-RT-F*	CCA GAT GTG ATC ACC CTG TTG GAA CAA G	116
	Zfp30-RT-R	GGA AGT AAC CCG TTA CTG CTG TAT CTG GAT	

Table S2. **List of genes showing upregulated expression in Trpc3-expressing vs. Trpc3-deficient M1 macrophages.**

Gene ID	Fold change	Gene Symbol	Entrez ID	Mmu	Start	End	Strand	Gene Type
ENSMUSG00000055494	3.4049115	*Gm14168*		chr2	156571792	156577457	+	lincRNA
ENSMUSG00000066158	2.6719732	*AY512931*		chr8	45060702	45065418	-	lincRNA
ENSMUSG00000087252	2.36036	*Gm14379*		chrX	7375925	7378042	+	lincRNA
ENSMUSG00000064246	10.538905	*Chi3l1*	12654	chr1	134182176	134190181	+	protein_coding
ENSMUSG00000034795	4.5629654	*Ccdc122*	108811	chr14	77036772	77112257	+	protein_coding
ENSMUSG00000061062	4.4487205	*Gm10093*		chr17	78491565	78493541	+	protein_coding
ENSMUSG00000030532	4.333237	*Hddc3*	68695	chr7	80343137	80346097	+	protein_coding
ENSMUSG00000048647	4.2417855	*Exd1*	241624	chr2	119516505	119547627	-	protein_coding
ENSMUSG00000022501	4.0189557	*Prm1*	19118	chr16	10796326	10796886	-	protein_coding
ENSMUSG00000026390	3.9138236	*Marco*	17167	chr1	120474538	120505084	-	protein_coding
ENSMUSG00000022584	3.8905606	*Ly6c2*	1E+08	chr15	75108160	75111684	-	protein_coding
ENSMUSG00000021707	3.6582189	*Dhfr*	13361	chr13	92354783	92389053	+	protein_coding
ENSMUSG00000047473	3.6172707	*Zfp30*	22693	chr7	29783998	29794702	+	protein_coding
ENSMUSG00000079018	3.5189233	*Ly6c1*	17067	chr15	75045017	75048837	-	protein_coding
ENSMUSG00000056665	3.3343542	*4930572J05Rik*	223626	chr15	74721234	74724373	+	protein_coding
ENSMUSG00000064141	3.3215115	*Zfp69*	381549	chr4	120930137	120951699	-	protein_coding
ENSMUSG00000024730	3.265462	*Ms4a8a*	64381	chr19	11067472	11081103	-	protein_coding
ENSMUSG00000053719	3.1723056	*Klk1b26*		chr7	44012678	44016965	+	protein_coding
ENSMUSG00000026073	3.1661649	*Il1r2*	16178	chr1	40084768	40125219	+	protein_coding
ENSMUSG00000051065	3.1312573	*Mb21d2*	239796	chr16	28826176	28929698	-	protein_coding
ENSMUSG00000022629	3.1085415	*Kif21a*	16564	chr15	90933276	91049948	-	protein_coding
ENSMUSG00000008734	3.085958	*Gprc5b*	64297	chr7	118972040	118995211	-	protein_coding
ENSMUSG00000043068	3.0225008	*Fam89a*	69627	chr8	124740257	124751809	-	protein_coding
ENSMUSG00000029648	2.9262915	*Flt1*	14254	chr5	147561604	147726011	-	protein_coding
ENSMUSG00000024675	2.8419793	*Ms4a4c*	64380	chr19	11404770	11427246	+	protein_coding
ENSMUSG00000063713	2.8357823	*Klk1b24*	16617	chr7	44188236	44192452	+	protein_coding
ENSMUSG00000079339	2.758999	*Gm14446*	667373	chr19	34592888	34601968	-	protein_coding
ENSMUSG00000043424	2.6912928	*Eif3j2*	1E+08	chr18	43475418	43477836	-	protein_coding
ENSMUSG00000022099	2.690401	*Epb4.9*	13829	chr14	70602184	70636048	-	protein_coding
ENSMUSG00000058470	2.68049	*Gm8369*	666926	chr19	11492038	11512577	+	protein_coding
ENSMUSG00000066513	2.6640224	*Klk1b4*	18048	chr7	44207435	44211754	+	protein_coding
ENSMUSG00000027199	2.5935123	*Gatm*	67092	chr2	122594467	122611303	-	protein_coding
ENSMUSG00000071715	2.561802	*Ncf4*	17972	chr15	78244801	78262580	+	protein_coding

ENSMUSG00000028549	2.5495584	*Itgb3bp*	67733	chr4	99765402	99929813	-	protein_coding
ENSMUSG00000067297	2.4768102	*2010002M12Rik*	112419	chr19	34617049	34640743	-	protein_coding
ENSMUSG00000069516	2.459303	*Lyz2*	17105	chr10	117277334	117282274	-	protein_coding
ENSMUSG00000080725	2.44949	*Gm6121*	619991	chrX	26912743	26935697	-	protein_coding
ENSMUSG00000069515	2.4288287	*Lyz1*	17110	chr10	117287796	117292868	-	protein_coding
ENSMUSG00000006313	2.3801022	*Upk1a*	109637	chr7	30603092	30612734	-	protein_coding
ENSMUSG00000025491	2.3532352	*Ifitm1*	68713	chr7	140967221	140969825	+	protein_coding
ENSMUSG00000071788	2.3502622	*Gm14525*	1E+08	chrX	26672777	26702638	-	protein_coding
ENSMUSG00000079017	2.3390672	*Ifi27l2a*	76933	chr12	103442167	103443680	-	protein_coding
ENSMUSG00000027562	2.338534	*Car2*	12349	chr3	14886426	14900769	+	protein_coding
ENSMUSG00000046380	2.3365693	*Jrk*	16469	chr15	74702412	74709535	-	protein_coding
ENSMUSG00000053168	2.3347254	*9030619P08Rik*	105892	chr15	75427607	75431829	-	protein_coding
ENSMUSG00000003032	2.304764	*Klf4*	16600	chr4	55527143	55532466	-	protein_coding
ENSMUSG00000016356	2.284306	*Col20a1*	73368	chr2	180986535	181017540	+	protein_coding
ENSMUSG00000022257	2.2361846	*Laptm4b*	114128	chr15	34238026	34284295	+	protein_coding
ENSMUSG00000079625	2.201147	*Tm4sf19*	277203	chr16	32400506	32408227	+	protein_coding
ENSMUSG00000001520	2.1673348	*Nrip2*	60345	chr6	128399296	128408932	+	protein_coding
ENSMUSG00000036766	2.131711	*Dner*	227325	chr1	84369841	84696221	-	protein_coding
ENSMUSG00000096620	2.1278296	*Gm5169*	382277	chrX	25277605	25301455	-	protein_coding
ENSMUSG00000067889	2.0781372	*Spnb3*	20743	chr19	4711208	4752353	+	protein_coding
ENSMUSG00000018166	2.0521176	*Erbb3*	13867	chr10	128567523	128589652	-	protein_coding
ENSMUSG00000096768	2.0327523	*ERDR1*	170942	chrY	90784738	90816464	+	protein_coding
ENSMUSG00000022696	2.0245006	*Sidt1*	320007	chr16	44240180	44333196	-	protein_coding
ENSMUSG00000053101	2.018341	*Gpr141*	353346	chr13	19749682	19824257	-	protein_coding
ENSMUSG00000016526	2.0163202	*Dyrk3*	226419	chr1	131128441	131138234	-	protein_coding
ENSMUSG00000083670	2.3577778	*Gm6829*		chrX	9327749	9328108	-	pseudogene
ENSMUSG00000095526	2.0683854	*Gm10243*		chr6	48523456	48523885	+	pseudogene
NEWGENE60	2.9014115	*NEWGENE60*		chr8	15519774	15520124		
NEWGENE126	2.0652552	*NEWGENE126*		chr13	103591399	103591625		

Table S3. List of genes showing downregulated expression in Trpc3-expressing vs. Trpc3-deficient M1 macrophages.

Gene ID	Fold change	Gene Symbol	Entrez ID	Mmu	End	Start	Strand	Gene Type
ENSMUSG00000078190	2.3114076	*Dnm3os*		chr1	162225550	162217623	+	antisense
ENSMUSG00000075463	2.5456507	*4930594M22Rik*		chr14	122937469	122913030	+	lincRNA
ENSMUSG00000097330	2.5055995	*AC020971.1*		chr18	37768242	37746559	-	lincRNA
ENSMUSG00000096972	2.2493274	*AL929563.1*		chr2	169888503	169633646	-	lincRNA
ENSMUSG00000075511	2.1868012	*1700001L05Rik*		chr15	83367282	83357526	-	lincRNA
ENSMUSG00000050390	17.104002	*C77080*	97130	chr4	129261404	129219578	-	protein_coding
ENSMUSG00000029814	9.809325	*Igf2bp3*	140488	chr6	49214954	49085221	-	protein_coding
ENSMUSG00000063415	6.8926888	*Cyp26b1*	232174	chr6	84593908	84571414	-	protein_coding
ENSMUSG00000041594	6.241111	*Tmtc4*	70551	chr14	122984035	122918971	-	protein_coding
ENSMUSG00000039601	5.976573	*Rcan2*	53901	chr17	44039516	43801851	+	protein_coding
ENSMUSG00000047592	5.6115775	*Nxpe5*		chr5	138253363	138225898	+	protein_coding
ENSMUSG00000028766	5.3064384	*Alpl*	11647	chr4	137796384	137741733	-	protein_coding
ENSMUSG00000027430	4.586026	*Dtd1*	66044	chr2	144768758	144599897	+	protein_coding
ENSMUSG00000009585	4.402954	*Apobec3*	80287	chr15	79915906	79891659	+	protein_coding
ENSMUSG00000057897	4.164553	*Camk2b*	12323	chr11	6066362	5969644	-	protein_coding
ENSMUSG00000033470	4.1015964	*Cysltr2*	70086	chr14	73049114	73029128	-	protein_coding
ENSMUSG00000034245	3.9937398	*Hdac11*	232232	chr6	91174692	91156665	+	protein_coding
ENSMUSG00000004698	3.9561045	*Hdac9*	79221	chr12	34528889	34371503	-	protein_coding
ENSMUSG00000000567	3.9051445	*Sox9*	20682	chr11	112787760	112782224	+	protein_coding
ENSMUSG00000022518	3.8048134	*4930562C15Rik*	78809	chr16	4867691	4835416	+	protein_coding
ENSMUSG00000019326	3.5944316	*Aoc3*	11754	chr11	101341938	101330605	+	protein_coding
ENSMUSG00000037239	3.5639577	*Spred3*	101809	chr7	29169841	29158829	-	protein_coding
ENSMUSG00000031493	3.4657283	*Ggn*	243897	chr7	29173933	29170220	+	protein_coding
ENSMUSG00000002831	3.4519348	*Plin4*	57435	chr17	56109802	56100591	-	protein_coding
ENSMUSG00000026768	3.3912625	*Itga8*	241226	chr2	12301920	12106632	-	protein_coding
ENSMUSG00000053117	3.381054	*E330013P04Rik*		chr19	60162260	60144689	+	protein_coding
ENSMUSG00000020593	3.3191278	*Lpin1*	14245	chr12	16589770	16535669	-	protein_coding
ENSMUSG00000020427	3.3011422	*Igfbp3*	16009	chr11	7213923	7206086	-	protein_coding
ENSMUSG00000023032	3.276219	*Slc4a8*	59033	chr15	100823968	100761747	+	protein_coding
ENSMUSG00000006651	3.2603571	*Aplp1*	11803	chr7	30445582	30434981	-	protein_coding
ENSMUSG00000016498	3.248904	*Pdcd1lg2*	58205	chr19	29471157	29410919	+	protein_coding
ENSMUSG00000032816	3.241164	*Igdcc4*	56741	chr9	65137940	65101486	+	protein_coding
ENSMUSG00000074505	3.1928678	*Fat3*	270120	chr9	16378231	15910205	-	protein_coding

ENSMUSG00000030032	3.1842134	*Wdr54*	75659	chr6	83156397	83149361	-	protein_coding
ENSMUSG00000016024	3.1447988	*Lbp*	16803	chr2	158332852	158306493	+	protein_coding
ENSMUSG00000025854	3.1413965	*Fam20c*	80752	chr5	138810077	138754514	+	protein_coding
ENSMUSG00000020268	3.1123486	*Lyrm7*	75530	chr11	54860916	54826866	-	protein_coding
ENSMUSG00000049598	3.0847738	*Vsig8*	240916	chr1	172563717	172555938	+	protein_coding
ENSMUSG00000061740	3.0640643	*Cyp2d22*	56448	chr15	82380260	82370528	-	protein_coding
ENSMUSG00000038463	3.0211618	*Olfml2b*	320078	chr1	170682789	170644532	+	protein_coding
ENSMUSG00000040564	2.90582	*Apoc1*	11812	chr7	19692658	19689484	-	protein_coding
ENSMUSG00000029096	2.8988945	*Htra3*	78558	chr5	35679782	35652041	-	protein_coding
ENSMUSG00000028339	2.8875773	*Col15a1*	12819	chr4	47313167	47208161	+	protein_coding
ENSMUSG00000036862	2.8622968	*Dchs1*	233651	chr7	105787654	105752989	-	protein_coding
ENSMUSG00000028197	2.8104095	*Col24a1*	71355	chr3	145552011	145292472	+	protein_coding
ENSMUSG00000051043	2.808078	*Gprc5c*	70355	chr11	114872617	114851152	+	protein_coding
ENSMUSG00000024501	2.7920341	*Dpysl3*	22240	chr18	43438286	43320979	-	protein_coding
ENSMUSG00000016150	2.7075396	*Tenm1*	23963	chrX	43429126	42527866	-	protein_coding
ENSMUSG00000004552	2.6866808	*Ctse*	13034	chr1	131675503	131638306	+	protein_coding
ENSMUSG00000018339	2.59821	*Gpx3*		chr11	54910377	54902453	+	protein_coding
ENSMUSG00000051934	2.5633044	*Spats2*	72572	chr15	99213206	99126578	+	protein_coding
ENSMUSG00000025932	2.5589588	*Eya1*	14048	chr1	14310200	14168954	-	protein_coding
ENSMUSG00000022793	2.4887316	*B4galt4*	56375	chr16	38769049	38742264	+	protein_coding
ENSMUSG00000026355	2.4421906	*Mcm6*	17219	chr1	128359656	128331591	-	protein_coding
ENSMUSG00000040084	2.4209569	*Bub1b*	12236	chr2	118641591	118598211	+	protein_coding
ENSMUSG00000017707	2.398862	*Serinc3*	26943	chr2	163645131	163623272	-	protein_coding
ENSMUSG00000042616	2.3867917	*Oscp1*		chr4	126092195	126058565	+	protein_coding
ENSMUSG00000046818	2.375446	*Ddit4l*	73284	chr3	137628333	137621612	+	protein_coding
ENSMUSG00000033083	2.3641222	*Tbc1d4*	210789	chr14	101609191	101442360	-	protein_coding
ENSMUSG00000053062	2.3491647	*Jam2*	67374	chr16	84825928	84774123	+	protein_coding
ENSMUSG00000029231	2.347712	*Pdgfra*	18595	chr5	75198204	75152291	+	protein_coding
ENSMUSG00000096140	2.3287227	*D730048J04Rik*	1E+08	chr17	43543639	43534174	-	protein_coding
ENSMUSG00000020810	2.3153434	*Cygb*	114886	chr11	116654313	116645595	-	protein_coding
ENSMUSG00000095029	2.3024812	*AC238811.1*	1.01E+08	chr13	120027011	120026439	-	protein_coding
ENSMUSG00000027611	2.2939112	*Procr*	19124	chr2	155755471	155751117	+	protein_coding
ENSMUSG00000002020	2.2682023	*Ltbp2*	16997	chr12	84876532	84783212	-	protein_coding
ENSMUSG00000073538	2.257463	*E330020D12Rik*		chr1	153408429	153404827	-	protein_coding

ENSMUSG00000022639	2.23272	*5330426P16Rik*		chr16	50732743	50727966	-	protein_coding
ENSMUSG00000026646	2.2298458	*Suv39h2*	64707	chr2	3475031	3455815	-	protein_coding
ENSMUSG00000047907	2.2116473	*Tshz2*	228911	chr2	170071816	169633013	+	protein_coding
ENSMUSG00000021391	2.2024438	*Cenpp*	66336	chr13	49652746	49464027	-	protein_coding
ENSMUSG00000040978	2.2015476	*Gm11992*	626870	chr11	9069356	9048594	+	protein_coding
ENSMUSG00000002797	2.1976557	*Ggct*	110175	chr6	54992950	54982580	-	protein_coding
ENSMUSG00000028789	2.1853423	*Adc*	242669	chr4	128962442	128930233	-	protein_coding
ENSMUSG00000035413	2.1782575	*Tmem98*	103743	chr11	80822033	80810175	+	protein_coding
ENSMUSG00000035258	2.15866	*Abi3bp*	320712	chr16	56690128	56477846	+	protein_coding
ENSMUSG00000039814	2.1352198	*Xkr5*	319581	chr8	18950975	18932729	-	protein_coding
ENSMUSG00000066258	2.1331694	*Trim12a*	76681	chr7	104315466	104299894	-	protein_coding
ENSMUSG00000001119	2.126367	*Col6a1*	12833	chr10	76726168	76708792	-	protein_coding
ENSMUSG00000074218	2.118654	*Cox7a1*	12865	chr7	30186028	30184171	+	protein_coding
ENSMUSG00000030465	2.111129	*Psd3*	234353	chr8	68062286	67689082	-	protein_coding
ENSMUSG00000048503	2.1088326	*Tmem136*	235300	chr9	43116570	43108653	-	protein_coding
ENSMUSG00000043162	2.0865662	*Pigy*	66459	chr6	57692078	57686576	-	protein_coding
ENSMUSG00000034758	2.0662465	*Tle6*	114606	chr10	81601073	81590904	-	protein_coding
ENSMUSG00000032113	2.0659866	*Chek1*	12649	chr9	36727065	36708482	-	protein_coding
ENSMUSG00000059173	2.0590768	*Pde1a*	18573	chr2	80129458	79834453	-	protein_coding
ENSMUSG00000042485	2.0563378	*Mustn1*	66175	chr14	30881608	30879257	+	protein_coding
ENSMUSG00000021097	2.0323384	*Clmn*	94040	chr12	104865076	104763114	-	protein_coding
ENSMUSG00000047434	2.028453	*AI480653*	268880	chr16	31081432	30955627	-	protein_coding
ENSMUSG00000031616	2.021818	*Ednra*	13617	chr8	77724464	77663031	-	protein_coding
ENSMUSG00000043587	2.019966	*Acpl2*	235534	chr9	96892669	96823344	-	protein_coding
ENSMUSG00000027323	2.0142841	*Rad51*	19361	chr2	119147445	119112793	+	protein_coding
ENSMUSG00000033213	2.0102997	*AA467197*	433470	chr2	122641191	122636986	+	protein_coding
ENSMUSG00000082953	2.0579538	*Gm13217*		chr2	9042484	9041117	-	pseudogene
ENSMUSG00000097656	2.0393302	*RP23-291E6.5*		chr6	57689649	57689433	-	pseudogene
ENSMUSG00000086291	2.589676	*Gm15513*		chr5	34213802	34211810	-	sense_intronic
ENSMUSG00000096832	14.456217	*SNORD93*		chr5	23711061	23710991	-	snoRNA
NEWGENE43	3.7216446	*NEWGENE43*		chr7	145188167	145183170		

Table S4. **Top ten genes with highest fold change in upregulated expression in Trpc3-expressing vs. Trpc3-deficient M1 macrophages.**

Gene ID	Fold Change	Gene Symbol	Mmu	Description
ENSMUSG00000064246	10.538905	*Chi3l1*	chr1	chitinase 3-like 1 [Source:MGI Symbol;Acc:MGI:1340899]
ENSMUSG00000034795	4.5629654	*Ccdc122*	chr14	coiled-coil domain containing 122 [Source:MGI Symbol;Acc:MGI:1918358]
ENSMUSG00000061062	4.4487205	*Gm10093*	chr17	predicted pseudogene 10093 [Source:MGI Symbol;Acc:MGI:3704479]
ENSMUSG00000030532	4.333237	*Hddc3*	chr7	HD domain containing 3 [Source:MGI Symbol;Acc:MGI:1915945]
ENSMUSG00000048647	4.2417855	*Exd1*	chr2	exonuclease 3'-5' domain containing 1 [Source:MGI Symbol;Acc:MGI:3045306]
ENSMUSG00000022501	4.0189557	*Prm1*	chr16	protamine 1 [Source:MGI Symbol;Acc:MGI:97765]
ENSMUSG00000026390	3.9138236	*Marco*	chr1	macrophage receptor with collagenous structure [Source:MGI Symbol;Acc:MGI:1309998]
ENSMUSG00000022584	3.8905606	*Ly6c2*	chr15	lymphocyte antigen 6 complex, locus C2 [Source:MGI Symbol;Acc:MGI:3712069]
ENSMUSG00000021707	3.6582189	*Dhfr*	chr13	dihydrofolate reductase [Source:MGI Symbol;Acc:MGI:94890]
ENSMUSG00000047473	3.6172707	*Zfp30*	chr7	zinc finger protein 30 [Source:MGI Symbol;Acc:MGI:99178]

Table S5. **Top ten genes with highest fold change in downregulated expression in Trpc3-expressing vs. Trpc3-deficient M1 macrophages.**

Gene ID	Fold change	Gene symbol	Mmu	Description
ENSMUSG00000050390	17.104	*C77080*	chr4	expressed sequence C77080 [Source:MGI Symbol;Acc:MGI:2140651]
ENSMUSG00000029814	9.809325	*Igf2bp3*	chr6	insulin-like growth factor 2 mRNA binding protein 3 [Source:MGI Symbol;Acc:MGI:1890359]
ENSMUSG00000063415	6.892689	Cyp26b1	chr6	cytochrome P450, family 26, subfamily b, polypeptide 1 [Source:MGI Symbol;Acc:MGI:2176159]
ENSMUSG00000041594	6.241111	*Tmtc4*	chr14	transmembrane and tetratricopeptide repeat containing 4 [Source:MGI Symbol;Acc:MGI:1921050]
ENSMUSG00000039601	5.976573	*Rcan2*	chr17	regulator of calcineurin 2 [Source:MGI Symbol;Acc:MGI:1858219]
ENSMUSG00000047592	5.611578	*Nxpe5*	chr5	neurexophilin and PC-esterase domain family, member 5 [Source:MGI Symbol;Acc:MGI:3584036]
ENSMUSG00000028766	5.306438	*Alpl*	chr4	alkaline phosphatase, liver/bone/kidney [Source:MGI Symbol;Acc:MGI:87983]
ENSMUSG00000027430	4.586026	*Dtd1*	chr2	D-tyrosyl-tRNA deacylase 1 homolog (S. cerevisiae) [Source:MGI Symbol;Acc:MGI:1913294]
ENSMUSG00000009585	4.402954	*Apobec3*	chr15	apolipoprotein B mRNA editing enzyme, catalytic polypeptide 3 [Source:MGI Symbol;Acc:MGI:1933111]
ENSMUSG00000057897	4.164553	*Camk2b*	chr11	calcium/calmodulin-dependent protein kinase II, beta [Source:MGI Symbol;Acc:MGI:88257]

Table S6. **Novel genes detected in M1 macrophages.**

Gene ID	Gene Symbol	Mmu	End	Start
NEWGENE32	NEWGENE32	chr1	30770795	30770672
NEWGENE33	NEWGENE33	chr1	48089525	48089309
NEWGENE34	NEWGENE34	chr1	51509137	51508988
NEWGENE35	NEWGENE35	chr1	88551721	88551559
NEWGENE36	NEWGENE36	chr1	102628340	102628132
NEWGENE37	NEWGENE37	chr1	122357335	122357127
NEWGENE38	NEWGENE38	chr1	131574045	131573786
NEWGENE39	NEWGENE39	chr1	151243103	151240183
NEWGENE40	NEWGENE40	chr1	154050829	154050672
NEWGENE41	NEWGENE41	chr1	185497866	185497407
NEWGENE73	NEWGENE73	chr2	13851234	13850803
NEWGENE72	NEWGENE72	chr2	45282798	45279043
NEWGENE74	NEWGENE74	chr2	109058229	109057636
NEWGENE13	NEWGENE13	chr3	5860826	5860537
NEWGENE14	NEWGENE14	chr3	8462980	8461387
NEWGENE15	NEWGENE15	chr3	14376320	14369852
NEWGENE16	NEWGENE16	chr3	23939362	23939218
NEWGENE17	NEWGENE17	chr3	38427207	38426552
NEWGENE18	NEWGENE18	chr3	48026637	48025956
NEWGENE19	NEWGENE19	chr3	50010172	50009519
NEWGENE20	NEWGENE20	chr3	50517257	50516601
NEWGENE21	NEWGENE21	chr3	52173753	52173585
NEWGENE22	NEWGENE22	chr3	62159405	62159046
NEWGENE23	NEWGENE23	chr3	92176590	92173998
NEWGENE24	NEWGENE24	chr3	112081540	112081393
NEWGENE25	NEWGENE25	chr3	121383699	121382176
NEWGENE26	NEWGENE26	chr3	127952161	127951901
NEWGENE27	NEWGENE27	chr3	145677740	145677465
NEWGENE28	NEWGENE28	chr3	146368599	146367997
NEWGENE29	NEWGENE29	chr4	23101611	23093713
NEWGENE30	NEWGENE30	chr4	40889822	40889626
NEWGENE31	NEWGENE31	chr4	142780775	142779539
NEWGENE76	NEWGENE76	chr5	23385169	23384143
NEWGENE77	NEWGENE77	chr5	29888766	29888640
NEWGENE75	NEWGENE75	chr5	31571447	31569617
NEWGENE78	NEWGENE78	chr5	67469412	67469004
NEWGENE79	NEWGENE79	chr5	101471102	101469948
NEWGENE80	NEWGENE80	chr5	104584455	104582434
NEWGENE81	NEWGENE81	chr5	151431105	151430898
NEWGENE5	NEWGENE5	chr6	3201623	3201428
NEWGENE1	NEWGENE1	chr6	3346069	3330794
NEWGENE6	NEWGENE6	chr6	47432133	47431798
NEWGENE2	NEWGENE2	chr6	47752919	47654921
NEWGENE7	NEWGENE7	chr6	69111206	69110911
NEWGENE8	NEWGENE8	chr6	72015640	72015003
NEWGENE9	NEWGENE9	chr6	79034946	79034475

NEWGENE10	NEWGENE10	chr6	82804385	82804165
NEWGENE11	NEWGENE11	chr6	105492659	105482365
NEWGENE3	NEWGENE3	chr6	124581044	124571356
NEWGENE12	NEWGENE12	chr6	131199789	131199072
NEWGENE4	NEWGENE4	chr6	134316330	134310356
NEWGENE44	NEWGENE44	chr7	16049066	16048392
NEWGENE45	NEWGENE45	chr7	17287726	17287588
NEWGENE46	NEWGENE46	chr7	39572770	39563684
NEWGENE47	NEWGENE47	chr7	40850253	40849854
NEWGENE48	NEWGENE48	chr7	48137595	48137432
NEWGENE49	NEWGENE49	chr7	48913065	48912860
NEWGENE50	NEWGENE50	chr7	59322831	59322497
NEWGENE51	NEWGENE51	chr7	70273976	70273479
NEWGENE52	NEWGENE52	chr7	73686249	73685579
NEWGENE53	NEWGENE53	chr7	75036463	75036181
NEWGENE54	NEWGENE54	chr7	89431290	89431162
NEWGENE55	NEWGENE55	chr7	97788764	97788546
NEWGENE56	NEWGENE56	chr7	100679803	100678985
NEWGENE57	NEWGENE57	chr7	106639114	106637281
NEWGENE58	NEWGENE58	chr7	111119891	111119707
NEWGENE42	NEWGENE42	chr7	129566351	129559710
NEWGENE43	NEWGENE43	chr7	145188167	145183170
NEWGENE60	NEWGENE60	chr8	15520124	15519774
NEWGENE61	NEWGENE61	chr8	25303547	25303415
NEWGENE62	NEWGENE62	chr8	27297148	27296316
NEWGENE63	NEWGENE63	chr8	28084808	28083198
NEWGENE64	NEWGENE64	chr8	33485751	33484314
NEWGENE65	NEWGENE65	chr8	80950710	80950552
NEWGENE66	NEWGENE66	chr8	96512701	96512364
NEWGENE67	NEWGENE67	chr8	109340906	109339800
NEWGENE68	NEWGENE68	chr8	128173556	128166400
NEWGENE69	NEWGENE69	chr8	128252168	128223061
NEWGENE70	NEWGENE70	chr8	128297983	128297833
NEWGENE71	NEWGENE71	chr8	128341757	128341563
NEWGENE59	NEWGENE59	chr8	129282911	129269609
NEWGENE96	NEWGENE96	chr9	3259132	3258789
NEWGENE97	NEWGENE97	chr9	40052600	40050707
NEWGENE98	NEWGENE98	chr9	41680437	41680216
NEWGENE99	NEWGENE99	chr9	48420225	48420066
NEWGENE100	NEWGENE100	chr9	52003321	52002015
NEWGENE101	NEWGENE101	chr9	54824930	54824459
NEWGENE102	NEWGENE102	chr9	65535091	65534930
NEWGENE103	NEWGENE103	chr9	71019094	71018211
NEWGENE104	NEWGENE104	chr9	98748827	98748599
NEWGENE105	NEWGENE105	chr9	110281381	110281245
NEWGENE106	NEWGENE106	chr9	113497810	113497583
NEWGENE107	NEWGENE107	chr9	123928543	123927827

NEWGENE108	NEWGENE108	chr9	124269886	124268799
NEWGENE109	NEWGENE109	chr9	124476909	124474702
NEWGENE131	NEWGENE131	chr10	3198585	3198321
NEWGENE132	NEWGENE132	chr10	7388959	7388706
NEWGENE133	NEWGENE133	chr10	8548947	8544928
NEWGENE134	NEWGENE134	chr10	10614259	10612977
NEWGENE135	NEWGENE135	chr10	11492266	11492058
NEWGENE136	NEWGENE136	chr10	19207711	19207566
NEWGENE137	NEWGENE137	chr10	26674651	26673450
NEWGENE138	NEWGENE138	chr10	44689251	44688358
NEWGENE139	NEWGENE139	chr10	51531187	51526575
NEWGENE140	NEWGENE140	chr10	53499387	53498974
NEWGENE141	NEWGENE141	chr10	65944720	65939794
NEWGENE130	NEWGENE130	chr10	81880884	81878057
NEWGENE142	NEWGENE142	chr10	87968017	87966944
NEWGENE143	NEWGENE143	chr10	95739353	95739153
NEWGENE144	NEWGENE144	chr10	117925665	117925481
NEWGENE145	NEWGENE145	chr10	122566715	122565066
NEWGENE146	NEWGENE146	chr10	124524367	124523479
NEWGENE175	NEWGENE175	chr11	49999136	49998992
NEWGENE84	NEWGENE84	chr12	11882678	11882195
NEWGENE85	NEWGENE85	chr12	17900786	17900660
NEWGENE82	NEWGENE82	chr12	18065609	18061969
NEWGENE86	NEWGENE86	chr12	20241715	20241589
NEWGENE83	NEWGENE83	chr12	23115547	23111964
NEWGENE87	NEWGENE87	chr12	23150451	23150325
NEWGENE88	NEWGENE88	chr12	28438454	28437958
NEWGENE89	NEWGENE89	chr12	31020352	31020032
NEWGENE90	NEWGENE90	chr12	51134993	51134850
NEWGENE91	NEWGENE91	chr12	67668639	67658565
NEWGENE92	NEWGENE92	chr12	74346386	74346229
NEWGENE93	NEWGENE93	chr12	96651641	96650069
NEWGENE94	NEWGENE94	chr12	99645524	99645389
NEWGENE95	NEWGENE95	chr12	111013531	111013357
NEWGENE113	NEWGENE113	chr13	6957971	6956642
NEWGENE114	NEWGENE114	chr13	15815385	15814748
NEWGENE115	NEWGENE115	chr13	37589326	37581056
NEWGENE116	NEWGENE116	chr13	49169950	49169417
NEWGENE110	NEWGENE110	chr13	61786549	61780603
NEWGENE111	NEWGENE111	chr13	62236142	62203432
NEWGENE117	NEWGENE117	chr13	67954167	67953963
NEWGENE118	NEWGENE118	chr13	67969928	67969659
NEWGENE119	NEWGENE119	chr13	67985660	67985416
NEWGENE112	NEWGENE112	chr13	68450077	68233965
NEWGENE120	NEWGENE120	chr13	74557083	74552055
NEWGENE121	NEWGENE121	chr13	74605648	74605321
NEWGENE122	NEWGENE122	chr13	90180034	90179217

NEWGENE123	NEWGENE123	chr13	97378942	97378808
NEWGENE124	NEWGENE124	chr13	97583298	97583142
NEWGENE125	NEWGENE125	chr13	102659654	102659369
NEWGENE126	NEWGENE126	chr13	103591625	103591399
NEWGENE127	NEWGENE127	chr14	19278286	19277945
NEWGENE128	NEWGENE128	chr14	73715342	73713799
NEWGENE129	NEWGENE129	chr14	114940618	114940417
NEWGENE166	NEWGENE166	chr15	18369627	18369425
NEWGENE167	NEWGENE167	chr15	60144650	60144270
NEWGENE168	NEWGENE168	chr15	61288801	61288441
NEWGENE169	NEWGENE169	chr15	74935817	74934479
NEWGENE170	NEWGENE170	chr15	75075026	75071081
NEWGENE165	NEWGENE165	chr15	75135170	75130859
NEWGENE171	NEWGENE171	chr15	100657182	100652318
NEWGENE147	NEWGENE147	chr16	19065699	19061838
NEWGENE148	NEWGENE148	chr16	70999060	70998893
NEWGENE149	NEWGENE149	chr16	81781743	81780433
NEWGENE150	NEWGENE150	chr16	90146824	90146627
NEWGENE151	NEWGENE151	chr16	93132394	93121943
NEWGENE152	NEWGENE152	chr16	95184893	95184482
NEWGENE153	NEWGENE153	chr16	98064172	98062512
NEWGENE185	NEWGENE185	chr17	3258546	3258389
NEWGENE186	NEWGENE186	chr17	3309114	3308992
NEWGENE187	NEWGENE187	chr17	4082995	4082169
NEWGENE184	NEWGENE184	chr17	17205158	17202695
NEWGENE188	NEWGENE188	chr17	22288842	22286390
NEWGENE189	NEWGENE189	chr17	24781276	24779776
NEWGENE190	NEWGENE190	chr17	29435261	29435102
NEWGENE191	NEWGENE191	chr17	43155280	43150702
NEWGENE192	NEWGENE192	chr17	73259437	73259200
NEWGENE193	NEWGENE193	chr17	81670179	81669991
NEWGENE194	NEWGENE194	chr17	84049486	84041888
NEWGENE195	NEWGENE195	chr17	84160091	84159945
NEWGENE156	NEWGENE156	chr18	13041462	13040046
NEWGENE157	NEWGENE157	chr18	15214670	15206949
NEWGENE158	NEWGENE158	chr18	15430454	15430254
NEWGENE159	NEWGENE159	chr18	17235151	17234513
NEWGENE160	NEWGENE160	chr18	34047255	34047124
NEWGENE161	NEWGENE161	chr18	35001650	35001520
NEWGENE162	NEWGENE162	chr18	66509528	66509364
NEWGENE163	NEWGENE163	chr18	68692317	68692063
NEWGENE164	NEWGENE164	chr18	85698291	85697923
NEWGENE155	NEWGENE155	chr18	90592420	90588951
NEWGENE176	NEWGENE176	chr19	8033566	8019581
NEWGENE177	NEWGENE177	chr19	11388461	11375476
NEWGENE178	NEWGENE178	chr19	21162122	21161935
NEWGENE179	NEWGENE179	chr19	41011483	41011342

NEWGENE180	NEWGENE180	chr19	52947196	52946735
NEWGENE181	NEWGENE181	chr19	54258928	54258596
NEWGENE182	NEWGENE182	chr19	59558581	59558430
NEWGENE183	NEWGENE183	chr19	60079950	60079792
NEWGENE172	NEWGENE172	chrX	5446633	5446495
NEWGENE173	NEWGENE173	chrX	26414869	26414585
NEWGENE174	NEWGENE174	chrX	45607027	45606904
NEWGENE154	NEWGENE154	chrY	679645	670629

Table S7. **List of transcripts showing differential splicing in Trpc3-expressing vs. Trpc3-deficient M1 macrophages.**

Gene ID	Splicing Index	Transcript	Gene Symb	Entrez ID	Mmu	End	Start	Strand	Gene Type
ENSMUSG00000004552	0.5795363	Novel	Ctse	13034	chr1	1.32E+08	1.32E+08	+	protein_coding
ENSMUSG00000033852	0.4810525	ENSMUST00000143794	Pla2g4b		chr2	1.2E+08	1.2E+08	+	protein_coding
ENSMUSG00000005804	0.30251062	ENSMUST00000005954	Bloc1s6	18457	chr2	1.23E+08	1.23E+08	+	protein_coding
ENSMUSG00000027932	0.26242062	ENSMUST00000132041	Slc27a3	26568	chr3	90389938	90385239	-	protein_coding
ENSMUSG00000028289	0.6643897	ENSMUST00000108191	Epha7	13841	chr4	28967499	28813131	+	protein_coding
ENSMUSG00000028339	0.42727545	ENSMUST00000102917	Col15a1	12819	chr4	47313167	47208161	+	protein_coding
ENSMUSG00000003411	0.27447766	ENSMUST00000003502	Rab3b	69908	chr4	1.09E+08	1.09E+08	+	protein_coding
ENSMUSG00000064141	0.34895456	ENSMUST00000106281	Zfp69	381549	chr4	1.21E+08	1.21E+08	-	protein_coding
ENSMUSG00000060572	0.27997872	ENSMUST00000071977	Mfap2	17150	chr4	1.41E+08	1.41E+08	+	protein_coding
ENSMUSG00000038295	0.29514098	ENSMUST00000138716	Atg9b	213948	chr5	24392143	24384181	-	protein_coding
ENSMUSG00000025855	0.3722213	ENSMUST00000026973	Prkar1b	19085	chr5	1.39E+08	1.39E+08	-	protein_coding
ENSMUSG00000030587	0.50819206	ENSMUST00000151339	2200002D(	72275	chr7	29248432	29246561	-	protein_coding
ENSMUSG00000060441	0.39040262	ENSMUST00000138557	Trim5	667823	chr7	1.04E+08	1.04E+08	-	protein_coding
ENSMUSG00000014782	0.28100038	ENSMUST00000161672	Plekhg4	102075	chr8	1.05E+08	1.05E+08	+	protein_coding
ENSMUSG00000079055	0.31133842	ENSMUST00000085238	Slc8a3	110893	chr12	81333180	81197915	-	protein_coding
ENSMUSG00000021097	0.2741915	ENSMUST00000109936	Clmn	94040	chr12	1.05E+08	1.05E+08	-	protein_coding
ENSMUSG00000022099	0.32147986	ENSMUST00000022694	Epb4.9	13829	chr14	70636048	70602184	-	protein_coding
ENSMUSG00000022696	0.5005714	ENSMUST00000127567	Sidt1	320007	chr16	44333196	44240180	-	protein_coding
ENSMUSG00000055945	0.62096196	ENSMUST00000163887	Prr18	320111	chr17	8344112	8340406	+	protein_coding
ENSMUSG00000039601	0.4923949	ENSMUST00000044895	Rcan2	53901	chr17	44039516	43801851	+	protein_coding
ENSMUSG00000031099	0.25065482	ENSMUST00000088973	Smarca1	93761	chrX	47892974	47809368	-	protein_coding

<u>Table S8.</u> Top ten GEO enriched biological processes based on differentially expressed transcripts in Trpc3-expressing vs. Trpc3-deficient M1 macrophages.

For each GO category, the first row lists sub-root (biological process, molecular function, or cellular component), and corresponding GO ID. The second row lists number of reference genes in the category (C), number of genes in the gene set and also in the category (O), expected number in thecategory (E), Ratio of enrichment (R), p value from hypergeometric test (rawP), and p value adjusted by the multiple test adjustment (adjP).Finally, genes in the category are listed. For each gene, the table lists the user uploaded ID, symbol, and description.

**biological process**	**cell migration**	**GO:0030334**
C=399;O=12;E=2.17;R=5.52;rawP=1.98e-06;adjP=0.0013		
ENSMUSG00000024501	*Dpysl3*	dihydropyrimidinase-like 3
ENSMUSG00000096768	*Erdr1*	erythroid differentiation regulator 1
ENSMUSG00000029648	*Flt1*	FMS-like tyrosine kinase 1
ENSMUSG00000031616	*Ednra*	endothelin receptor type A
ENSMUSG00000003032	*Klf4*	Kruppel-like factor 4 (gut)
ENSMUSG00000004698	*Hdac9*	histone deacetylase 9
ENSMUSG00000016024	*Lbp*	lipopolysaccharide binding protein
ENSMUSG00000019326	*Aoc3*	amine oxidase, copper containing 3
ENSMUSG00000000567	*Sox9*	SRY-box containing gene 9
ENSMUSG00000020427	*Igfbp3*	insulin-like growth factor binding protein 3
ENSMUSG00000025491	*Ifitm1*	interferon induced transmembrane protein 1
ENSMUSG00000029231	*Pdgfra*	platelet derived growth factor receptor a

**biological process**	**cell motility**	**GO:2000145**
C=415;O=12;E=2.26;R=5.31;rawP=2.97e-06;adjP=0.0013		
ENSMUSG00000024501	*Dpysl3*	dihydropyrimidinase-like 3
ENSMUSG00000096768	*Erdr1*	erythroid differentiation regulator 1
ENSMUSG00000029648	*Flt1*	FMS-like tyrosine kinase 1
ENSMUSG00000031616	*Ednra*	endothelin receptor type A
ENSMUSG00000003032	*Klf4*	Kruppel-like factor 4 (gut)

ENSMUSG00000004698	*Hdac9*	histone deacetylase 9
ENSMUSG00000016024	*Lbp*	lipopolysaccharide binding protein
ENSMUSG00000019326	*Aoc3*	amine oxidase, copper containing 3
ENSMUSG00000000567	*Sox9*	SRY-box containing gene 9
ENSMUSG00000020427	*Igfbp3*	insulin-like growth factor binding protein 3
ENSMUSG00000025491	*Ifitm1*	IFN induced transmembrane protein 1
ENSMUSG00000029231	*Pdgfra*	platelet derived growth factor receptor a

biological process **locomotion** **GO:0040012**

C=449;O=12;E=2.45;R=4.91;rawP=6.62e-06;adjP=0.0019

ENSMUSG00000024501	*Dpysl3*	dihydropyrimidinase-like 3
ENSMUSG00000096768	*Erdr1*	erythroid differentiation regulator 1
ENSMUSG00000029648	*Flt1*	FMS-like tyrosine kinase 1
ENSMUSG00000031616	*Ednra*	endothelin receptor type A
ENSMUSG00000003032	*Klf4*	Kruppel-like factor 4 (gut)
ENSMUSG00000004698	*Hdac9*	histone deacetylase 9
ENSMUSG00000016024	*Lbp*	lipopolysaccharide binding protein
ENSMUSG00000019326	*Aoc3*	amine oxidase, copper containing 3
ENSMUSG00000000567	*Sox9*	SRY-box containing gene 9
ENSMUSG00000020427	*Igfbp3*	insulin-like growth factor binding protein 3
ENSMUSG00000025491	*Ifitm1*	IFN induced transmembrane protein 1
ENSMUSG00000029231	*Pdgfra*	platelet derived growth factor receptor a

biological process **cellular movement** **GO:0051270**

C=462;O=12;E=2.52;R=4.77;rawP=8.83e-06;adjP=0.0019

ENSMUSG00000024501	*Dpysl3*	dihydropyrimidinase-like 3

ENSMUSG00000096768	*Erdr1*	erythroid differentiation regulator 1
ENSMUSG00000029648	*Flt1*	FMS-like tyrosine kinase 1
ENSMUSG00000031616	*Ednra*	endothelin receptor type A
ENSMUSG00000003032	*Klf4*	Kruppel-like factor 4 (gut)
ENSMUSG00000004698	*Hdac9*	histone deacetylase 9
ENSMUSG00000016024	*Lbp*	lipopolysaccharide binding protein
ENSMUSG00000019326	*Aoc3*	amine oxidase, copper containing 3
ENSMUSG00000000567	*Sox9*	SRY-box containing gene 9
ENSMUSG00000020427	*Igfbp3*	insulin-like growth factor binding protein 3
ENSMUSG00000025491	*Ifitm1*	IFN induced transmembrane protein 1
ENSMUSG00000029231	*Pdgfra*	platelet derived growth factor receptor a

biological process **PI3-kinase activity** **GO:0043551**
C=29;O=4;E=0.16;R=25.31;rawP=1.80e-05;adjP=0.0031

ENSMUSG00000003032	*Klf4*	Kruppel-like factor 4 (gut)
ENSMUSG00000066513	*Klk1b4*	kallikrein 1-related pepidase b4
ENSMUSG00000029231	*Pdgfra*	platelet derived growth factor receptor a
ENSMUSG00000029648	*Flt1*	FMS-like tyrosine kinase 1

biological process **lipid kinase activity** **GO:0043550**
C=34;O=4;E=0.19;R=21.59;rawP=3.44e-05;adjP=0.0050

ENSMUSG00000003032	*Klf4*	Kruppel-like factor 4 (gut)
ENSMUSG00000066513	*Klk1b4*	kallikrein 1-related pepidase b4
ENSMUSG00000029231	*Pdgfra*	platelet derived growth factor receptor a
ENSMUSG00000029648	*Flt1*	FMS-like tyrosine kinase 1

biological process **inositol lipid signaling** **GO:0048017**
C=83;O=5;E=0.45;R=11.06;rawP=9.18e-05;adjP=0.0087

ENSMUSG00000003032	*Klf4*	Kruppel-like factor 4 (gut)
ENSMUSG00000018166	*Erbb3*	v-erb erythroblastic leukemia viral oncogene
ENSMUSG00000000567	*Sox9*	SRY-box containing gene 9
ENSMUSG00000029231	*Pdgfra*	platelet derived growth factor receptor a
ENSMUSG00000029648	*Flt1*	FMS-like tyrosine kinase 1

biological process **ERK1 and ERK2 cascade** **GO:0070371**

C=137;O=6;E=0.75;R=8.04;rawP=0.0001;adjP=0.0087

ENSMUSG00000064246	*Chi3l1*	chitinase 3-like 1
ENSMUSG00000031616	*Ednra*	endothelin receptor type A
ENSMUSG00000003032	*Klf4*	Kruppel-like factor 4 (gut)
ENSMUSG00000033470	*Cysltr2*	cysteinyl leukotriene receptor 2
ENSMUSG00000000567	*Sox9*	SRY-box containing gene 9
ENSMUSG00000029231	*Pdgfra*	platelet derived growth factor receptor a

biological process **PI-signaling** **GO:0048015**

C=82;O=5;E=0.45;R=11.19;rawP=8.67e-05;adjP=0.0087

ENSMUSG00000003032	*Klf4*	Kruppel-like factor 4 (gut)
ENSMUSG00000018166	*Erbb3*	v-erb erythroblastic leukemia viral oncogene
ENSMUSG00000000567	*Sox9*	SRY-box containing gene 9
ENSMUSG00000029231	*Pdgfra*	platelet derived growth factor receptor a
ENSMUSG00000029648	*Flt1*	FMS-like tyrosine kinase 1

biological process **organic nitrogen** **GO:0010243**

C=437;O=10;E=2.38;R=4.20;rawP=0.0001;adjP=0.0087

ENSMUSG00000033083	*Tbc1d4*	TBC1 domain family, member 4
ENSMUSG00000031616	*Ednra*	endothelin receptor type A
ENSMUSG00000020593	*Lpin1*	lipin 1

ENSMUSG00000006651	*Aplp1*	amyloid beta (A4) precursor-like protein 1
ENSMUSG00000004698	*Hdac9*	histone deacetylase 9
ENSMUSG00000027199	*Gatm*	glycine amidinotransferase (L-arg:gly)
ENSMUSG00000001119	*Col6a1*	collagen, type VI, alpha 1
ENSMUSG00000000567	*Sox9*	SRY-box containing gene 9
ENSMUSG00000021707	*Dhfr*	dihydrofolate reductase
ENSMUSG00000029231	*Pdgfra*	platelet derived growth factor receptor a

Table S9. **Enriched KEGG pathways associated to transcripts showing downregulated expression in Trpc3-expressing vs. Trpc3-deficient M1 macrophages.**

For each pathway, the first row lists its KEGG pathway name, and corresponding KEGG ID. The second row lists number of reference genes in the category (C), number of genes in gene set and in the category (O), expected number in the category (E), Ratio of enrichment (R), p value from hypergeometric test (rawP), and p value adjusted by the multiple test adjustment (adjP).Finally, genes in the pathway are listed. For each gene, the table lists the user uploaded ID, gene symbol, and description.

**KEGG pathway**	**Calcium signaling**		**4020**
C=178;O=5;E=0.52;R=9.60;rawP=0.0002;adjP=0.0028			
ENSMUSG00000031616	*Ednra*	endothelin receptor type A	
ENSMUSG00000059173	*Pde1a*	phosphodiesterase 1A, cam-dependent	
ENSMUSG00000033470	*Cysltr2*	cysteinyl leukotriene receptor 2	
ENSMUSG00000057897	*Camk2b*	cam-dependent protein kinase II, beta	
ENSMUSG00000029231	*Pdgfra*	platelet derived growth factor receptor a	
**KEGG pathway**	**Cell adhesion molecules**		**4514**
C=149;O=3;E=0.44;R=6.88;rawP=0.0096;adjP=0.0385			
ENSMUSG00000026768	*Itga8*	integrin alpha 8	
ENSMUSG00000053062	*Jam2*	junction adhesion molecule 2	
ENSMUSG00000016498	*Pdcd1lg2*	programmed cell death 1 ligand 2	
**KEGG pathway**	**Cell cycle**		**4110**
C=127;O=3;E=0.37;R=8.08;rawP=0.0062;adjP=0.0385			
ENSMUSG00000026355	*Mcm6*	MIS5 homolog, S. pombe	
ENSMUSG00000032113	*Chek1*	checkpoint kinase 1	
ENSMUSG00000040084	*Bub1b*	budding uninhibited by benzimidazoles 1 homolog b	
**KEGG pathway**	**Glioma**		**5214**
C=66;O=2;E=0.19;R=10.36;rawP=0.0161;adjP=0.0385			
ENSMUSG00000057897	*Camk2b*	cam-dependent protein kinase II, beta	
ENSMUSG00000029231	*Pdgfra*	platelet derived growth factor receptor a	
**KEGG pathway**	**Focal adhesion**		**4510**

C=199;O=3;E=0.58;R=5.15;rawP=0.0207;adjP=0.0385

ENSMUSG00000026768	*Itga8*	integrin alpha 8
ENSMUSG00000001119	*Col6a1*	collagen, type VI, alpha 1
ENSMUSG00000029231	*Pdgfra*	platelet derived growth factor receptor a

KEGG pathway **Protein digestion/absorption** **4974**

C=78;O=2;E=0.23;R=8.77;rawP=0.0220;adjP=0.0385

ENSMUSG00000028339	*Col15a1*	collagen, type XV, alpha 1
ENSMUSG00000001119	*Col6a1*	collagen, type VI, alpha 1

KEGG pathway **p53 signaling** **4115**

C=69;O=2;E=0.20;R=9.91;rawP=0.0175;adjP=0.0385

ENSMUSG00000032113	*Chek1*	checkpoint kinase 1
ENSMUSG00000020427	*Igfbp3*	insulin-like growth factor binding protein 3

KEGG pathway **Glutathione metabolism** **480**

C=54;O=2;E=0.16;R=12.66;rawP=0.0110;adjP=0.0385

ENSMUSG00000018339	*Gpx3*	glutathione peroxidase 3
ENSMUSG00000002797	*Ggct*	gamma-glutamyl cyclotransferase

KEGG pathway **ECM-receptor interaction** **4512**

C=86;O=2;E=0.25;R=7.95;rawP=0.0264;adjP=0.0411

ENSMUSG00000026768	*Itga8*	integrin alpha 8
ENSMUSG00000001119	*Col6a1*	collagen, type VI, alpha 1

KEGG pathway **Actin cytoskeleton** **4810**

C=215;O=2;E=0.63;R=3.18;rawP=0.1308;adjP=0.1716

ENSMUSG00000026768	*Itga8*	integrin alpha 8
ENSMUSG00000029231	*Pdgfra*	platelet derived growth factor receptor a

Table S10. Enriched KEGG pathways associated to transcripts showing upregulated expression in Trpc3-expressing vs. Trpc3-deficient M1 macrophages.
For each pathway, the first row lists its KEGG pathway name, and corresponding KEGG ID. The second row lists number of reference genes in the category (C), number of genes in gene set and in the category (O), expected number in the category (E), Ratio of enrichment (R), p value from hypergeometric test (rawP), and p value adjusted by the multiple test adjustment (adjP).Finally, genes in the pathway are listed. For each gene, the table lists the user uploaded ID, gene symbol, and description.

**KEGG pathway**	**Salivary secretion**		**4970**
C=77;O=2;E=0.14;R=13.81;rawP=0.0093;adjP=0.0465			
ENSMUSG00000069516	*Lyz2*	lysozyme 2	
ENSMUSG00000069515	*Lyz1*	lysozyme 1	
**KEGG pathway**	**Cytokine-cytokine receptor interaction**		**4060**
C=245;O=2;E=0.46;R=4.34;rawP=0.0777;adjP=0.0971			
ENSMUSG00000026073	*Il1r2*	interleukin 1 receptor,type II	
ENSMUSG00000029648	*Flt1*	FMS-like tyrosine kinase 1	
**KEGG pathway**	**Endocytosis**		**4144**
C=219;O=2;E=0.41;R=4.86;rawP=0.0640;adjP=0.0971			
ENSMUSG00000018166	*Erbb3*	v-erb-b2 erythroblastic leukemia	
ENSMUSG00000029648	*Flt1*	FMS-like tyrosine kinase 1	
**KEGG pathway**	**Phagosome**		**4145**
C=176;O=2;E=0.33;R=6.04;rawP=0.0434;adjP=0.0971			
ENSMUSG00000026390	*Marco*	macrophage receptor collagenous structure	
ENSMUSG00000071715	*Ncf4*	neutrophil cytosolic factor 4	
**KEGG pathway**	**Metabolic pathways**		**1100**
C=1175;O=2;E=2.21;R=0.91;rawP=1.0000;adjP=1.0000			
ENSMUSG00000027199	*Gatm*	glycine amidinotransferase	
ENSMUSG00000021707	*Dhfr*	dihydrofolate reductase	

Supplemental figure 1. Evaluation of RNA quality by electrophoresis and bioanalyzer.

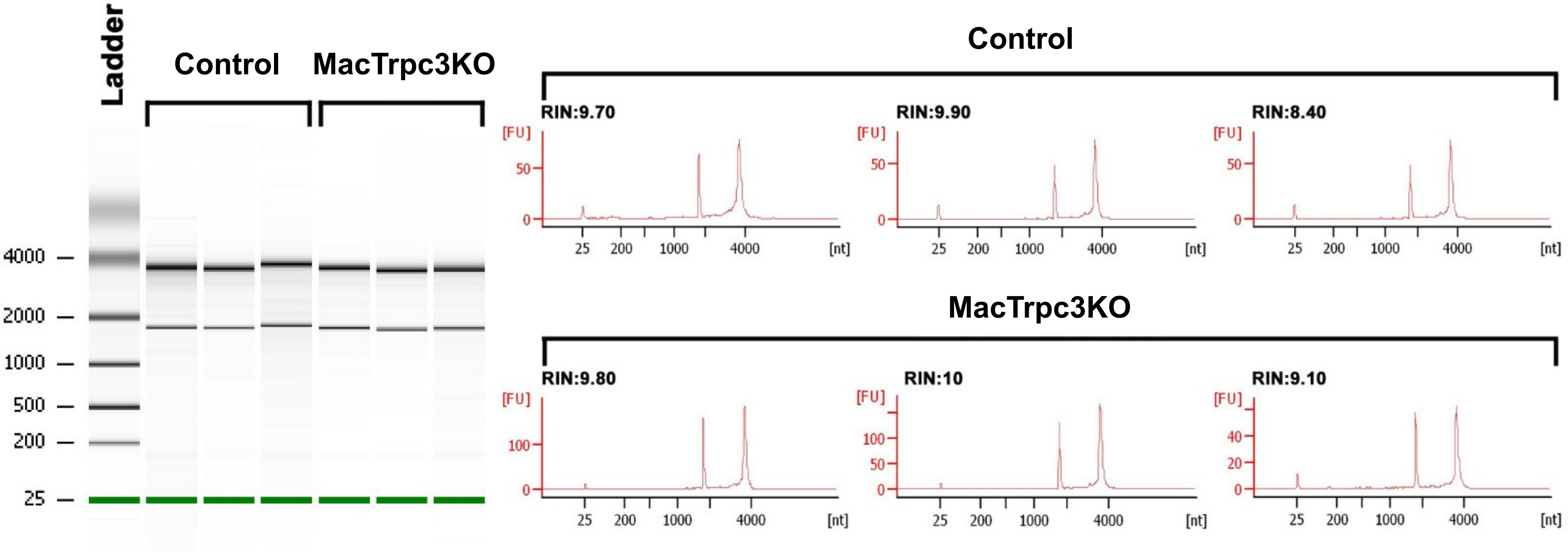

Supplement: Supplementary Information [file srep39867-s1.pdf]
